# Kir4.1 channels in NG2-glia play a role in development, potassium signaling, and ischemia-related myelin loss

**DOI:** 10.1038/s42003-018-0083-x

**Published:** 2018-06-28

**Authors:** Feier Song, Xiaoqi Hong, Jiayu Cao, Guofen Ma, Yanfei Han, Carlos Cepeda, Zizhen Kang, Tianle Xu, Shumin Duan, Jieqing Wan, Xiaoping Tong

**Affiliations:** 1 Discipline of Neuroscience and Department of Anatomy and Physiology, Shanghai Jiao Tong University School of Medicine, Shanghai, 200025 China; 2 Department of Neurosurgery, Shanghai Ren Ji Hospital, Shanghai, 200127 China; 3 Semel Institute for Neuroscience and Human Behavior, University of California Los Angeles School of Medicine, Los Angeles, 90024 CA USA; 4 Shanghai Institute of Immunology, Shanghai Jiao Tong University School of Medicine, Shanghai, 200025 China; 5 Department of Neurobiology, Key Laboratory of Medical Neurobiology of Ministry of Health of China, Zhejiang University School of Medicine, Hangzhou, 310058 China

## Abstract

The contribution of the inwardly rectifying K^+^ channel subtype Kir4.1 has been focused mainly on astrocytes, where they play important roles in the maintenance of resting membrane potential, extracellular K^+^ uptake, and facilitation of glutamate uptake in the central nervous system. Here, we report the role of Kir4.1 channels in NG2-glia during brain development, potassium signaling, and in an ischemic stroke disease model. Kir4.1 channels are widely expressed in NG2-glia during brain development. In the adult mouse hippocampus, Kir4.1 channels in NG2-glia constitute more than 80% of K^+^ channels inward currents. This large portion of Kir4.1 channel currents exhibits a deficit in NG2-glia as an initial response in a transient ischemic mouse model. Further evidence indicates that Kir4.1 deficits in NG2-glia potentially cause axonal myelin loss in ischemia through the association with oligodendrocyte-specific protein (OSP/Claudin-11), which unravels a potential therapeutic target in the treatment of ischemic stroke.

## Abstract (web summary)

Feier Song and colleagues have examined Kir4.1 channels in the mouse brain, and found global expression of functional channels during development. They also show that depletion of Kir4.1 channels impacts demyelination in ischemic stroke

## Introduction

The inwardly rectifying K^+^ channel subtype Kir4.1 has been well studied in astroglia within the central nervous system. Kir4.1 ion channels play prominent roles in the maintenance of resting membrane potential (RMP), extracellular K^+^ uptake, cell volume regulation, and facilitation of glutamate uptake, as well as in neurodegenerative diseases^1–6^. Although a pioneer developmental study indicated that Kir4.1 could be immunoactivated in NG2+ glial cells in rat optic nerve^7^, NG2-glia, which are known as oligodendrocyte precursor cells (OPCs), until recently were found to express high levels of *Kcnj10* gene (which encodes Kir4.1) in juvenile mouse brain, as evidenced by RNA-Seq transcriptome analysis^8,9^. However, whether NG2-glia functionally express Kir4.1 channels in the adult brain, as well as their newly found physiologic and/or pathologic relevance are largely unexplored^10,11^. Different from astrocytes, NG2-glia demonstrate self-renewal functionality as multipotent stem cells by providing myelinating oligodendrocytes during early brain development and receive direct synaptic contacts from both glutamatergic and GABAergic neurons^12–16^, suggesting that NG2-glia have much closer interactions with local neurons and greater impact on neural networks.

Stroke is a neural disease clinically manifested by transient or permanent brain dysfunction symptoms. As one of the three most common diseases in the world, stroke has a high mortality and disability rate. Ischemic stroke is the most common form, accounting for 87% of strokes and mainly causes impairment of neural cells and ultimately the loss of brain function due to ischemia and hypoxia. To date, treatment options are still limited^17^^,^^18^. Although there have been reports that glial cells contribute to the stroke pathology, the causes, disease mechanisms, and potential impacts remain unclear^11,19–23^. In the present study, we investigate the role of Kir4.1 channels in NG2-glia during brain development, potassium signaling, and in ischemia-related myelin loss. We demonstrate Kir4.1 channels in NG2-glia are widely expressed during brain development and constitute the bulk of K^+^ channel inward currents in adult hippocampus. Notably, in a transient middle cerebral artery occlusion (tMCAO) mouse model, Kir4.1 channel deficits in NG2-glia cause axonal myelin loss, thus unraveling a potential therapeutic target in the treatment of ischemic stroke.

## Results

### Expression of Kir4.1 in NG2-glia

**Fig. 1:**
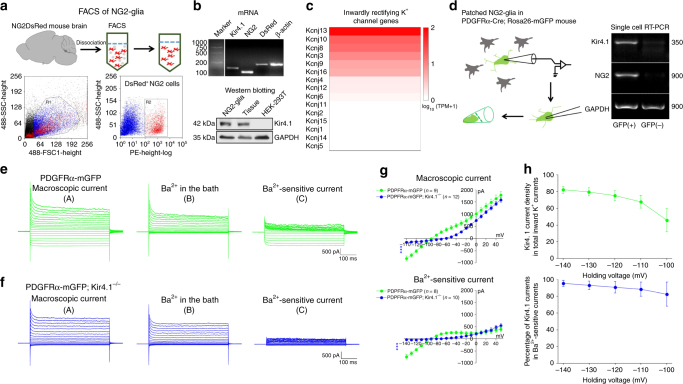
NG2-glia express potassium channel subtype Kir4.1 throughout mouse brain development. **a** The cartoon in the upper panel illustrates the approach for sorting tdTomato fluorescently labeled NG2 cells from NG2DsRedBAC transgenic mouse brain using the FACS method. Lower panel shows the brain cells harvested from postnatal 2-week-old NG2DsRedBAC mouse before and after sorting by FACS. Red fluorescently labeled cells are sorted NG2+ cells for sequential PCR and Western blot tests. **b** Representative PCR and Western blots showing Kir4.1 mRNA and protein expression in purified NG2-glia from NG2DsRedBAC transgenic mouse brain at postnatal 2 weeks. **c** The heat map shows a secondary enrichment gene kcnj10 (Kir4.1) among inwardly rectifying K^+^ channel genes family expressed in NG2-glia in a PDGFRα-creER; Rosa26-mGFP transgenic mouse brain at postnatal 7 weeks. **d** The cartoon and single-cell RT-PCR results illustrate single patched NG2-glia in PDGFRα-creERT; Rosa26-mGFP transgenic mouse hippocampus at postnatal 6–8 weeks. It clearly shows Kir4.1 expression in adult NG2-glia with GFP fluorescence identification. **e** Representative traces show macroscopic currents (A), macroscopic currents after bath application of Ba^2+^ (B), and Ba^2+^-sensitive currents (C) in a whole-cell patched NG2-glia in PDGFRα-mGFP transgenic mouse hippocampus at postnatal 8 weeks. *n* = 9 cells. **f** Representative traces showed the same as in (**e**) but for NG2-glia with Kir4.1 deletion from PDGFRα-mGFP; Kir4.1^−/−^ transgenic mouse at postnatal 8 weeks. *n* = 12 cells. **g** Average *I/V* plots showed a dramatic downregulation of inwardly rectifying current in NG2-glia from PDGFRα-mGFP; Kir4.1^−/−^ transgenic mice compared with its wild-type control. The error bars represent s.e.m. Statistical significance was assessed as indicated using two-tailed unpaired *t*-test. **h** The upper graph shows Kir4.1 currents in NG2-glia comprise a large portion of total inward K^+^ channel currents at a holding voltage of −140, −130, −120, −110, and −100 mV, respectively. The lower graph illustrates that more than 90% of Kir4.1 currents in NG2-glia are blocked by 100 µM Ba^2+^, which indicates a high efficiency of 100 µM Ba^2+^ pharmacological blockade for Kir4.1

To gain further insights into the role of Kir4.1 ion channels in NG2-glia, we undertook an investigation of these channels in a NG2DsRedBAC transgenic mouse strain. Fluorescent DsRed-labeled NG2-positive cells from postnatal 2-week-old mice were harvested and purified by fluorescence-activated cell sorting (FACS) (Fig. 1a, Supplementary Figure 1a). Both PCR and Western blot results illustrated that NG2-glia express Kir4.1 ion channel mRNA and protein (Fig. 1b), which is consistent with previous transcriptome and electrophysiological characterizations during early brain development^8,24^.

To investigate whether Kir4.1 channel expression persists during NG2-glia development, we further confirmed *Kcnj10* gene expression in adult NG2-glia using combined RNA-seq transcriptome and single-cell reverse transcriptase polymerase chain reaction (RT-PCR) techniques in PDGFRα-creERT; ROSA26-mGFP transgenic mouse brain, as this mouse strain displays a very high efficiency in labeling NG2-glia during brain development (Fig. 1c, d, Supplementary Figure 1b)^25,26^. Whole-cell patch recordings from single GFP-positive NG2-glial cells in acute hippocampal slices from 2- to 3-month-old PDGFRαCre-mGFP transgenic mice clearly demonstrated macroscopic K^+^ currents and Ba^2+^-sensitive currents^4^ (Fig. 1e). To accurately define the percentage of Kir4.1 channel-dependent currents involved in total inward K^+^ channel currents in adult NG2-glia, a tamoxifen-induced PDGFRαCre^ER^-mGFP; Kir4.1^f/f^ transgenic mouse was introduced to produce specific deletion of Kir4.1 in NG2-glia (Fig. 1f). Although no overt neurological deficits can be observed in Kir4.1 cKO mice at the examination time point P60, NG2-glia exhibited, on average, about 22.2 mV depolarization of the RMP and 7.5-fold increase of cell membrane resistance of GFP-labeled NG2-glia recorded in the hippocampal CA1 stratum radiatum (SR) compared with that in wild-type NG2-glia (Supplementary Figure 2). In addition, 82.2 ± 4.1% (*n* = 12) of Ba^2+^-sensitive currents in PDGFRα^+^ NG2-glia at a holding voltage of −140 mV was eliminated in Kir4.1 cKO mice (Fig. 1g, h), which further confirmed that Kir4.1 channel current contributes to a large portion of inward K^+^ channel currents in adult NG2-glia.

### Ischemia effects on Kir4.1 channels

**Fig. 2:**
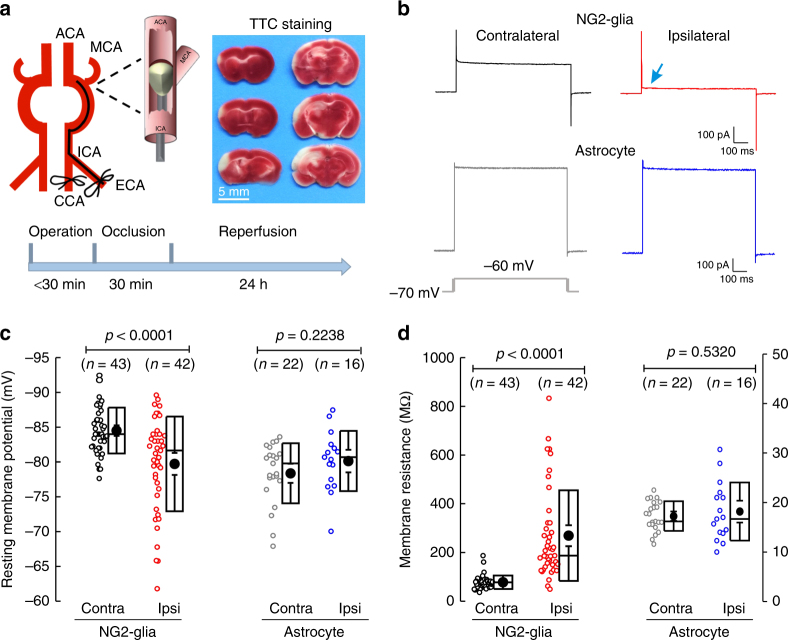
Membrane properties change in NG2-glia but not in astrocytes in a tMCAO mouse model. **a** The cartoon illustrates an established transient middle cerebral artery occlusion (tMCAO) mouse model of ischemic stroke. After MCA occlusion for 30 min, 2,3,5-triphenyltetrazolium chloride (TTC) staining was exhibited after 24 h reperfusion, as shown in the experimental diagram below. Right panel shows a series of brain sections obtained for TTC staining to detect the extent of tissue damage after 30 min of tMCAO. **b** Representative traces of whole-cell voltage-clamp recordings from NG2-glia and astrocytes in both contralateral and ipsilateral hippocampal CA1 regions after 30 min of tMCAO mice at postnatal 8 weeks. The current waveforms show the response to a 10 mV step depolarization, revealing clear differences between contralateral and ipsilateral NG2-glia. **c**, **d** Box plots summarize resting membrane potentials (**c**) and membrane resistances between −70 and −60 mV (**d**) for NG2-glia and astrocytes in both contralateral and ipsilateral hippocampal CA1 after 30 min of tMCAO. *n* indicates the cell numbers recorded. The data were normally distributed and statistical significance was assessed using two-tailed unpaired *t*-test, *P-*values are indicated

**Fig. 3:**
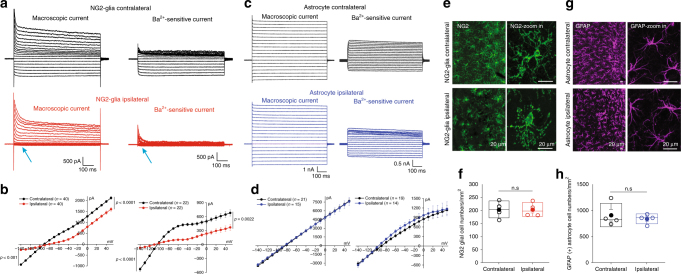
Kir4.1 channel deficits in NG2-glia but not in astrocytes in a tMCAO mouse model. **a** Representative traces show macroscopic currents and Ba^2+^-sensitive currents in NG2-glia in both contralateral (in black) and ipsilateral (in red) hippocampal CA1 regions of tMCAO mice at postnatal 8 weeks. The arrows show a dramatic loss of Ba^2+^-sensitive Kir4.1 current in ipsilateral NG2-glia. **b** Average *I/V* plots are for both macroscopic and Ba^2+^-sensitive currents in NG2-glia in tMCAO. The error bars represent s.e.m. *n* indicates the cell numbers recorded. Statistical significance was assessed using two-tailed unpaired *t*-test, *P-*values are indicated. **c** Representative traces show macroscopic currents and Ba^2+^-sensitive currents in astrocytes in both contralateral (in black) and ipsilateral (in blue) hippocampal CA1 regions of tMCAO mice at postnatal 8 weeks. There is no obvious change of macroscopic current and Ba^2+^-sensitive Kir4.1 current between contralateral and ipsilateral astrocytes in hippocampal CA1 regions of tMCAO. **d** Average *I*/*V* plots are for both macroscopic and Ba^2+^-sensitive currents in astrocytes in tMCAO. The error bars represent s.e.m. *n* indicates the cell numbers recorded. Statistical significance was assessed using two-tailed unpaired *t*-test. **e** Representative images show NG2 antibody-labeled positive cells in both contralateral and ipsilateral hippocampal CA1 regions of tMCAO mice at postnatal 8 weeks. Magnified images in right panels show ipsilateral NG2-glia reactive morphological changes compared with that in contralateral NG2-glia. Scale bars: 20 µm. **f** Bar graph shows NG2-glial cell numbers per mm^2^ in both sides of tMCAO. *n* = 4 mice. Statistical significance was assessed using two-tailed Mann–Whitney test. *P* > 0.05 as indicated. **g** Representative images show GFAP antibody-labeled positive cells in both contralateral and ipsilateral hippocampal CA1 regions of tMCAO mice at postnatal 8 weeks. Scale bars: 20 µm. **h** Bar graph shows GFAP + astroglia cell numbers per mm^2^ in both sides of tMCAO. *n* = 4 mice. *P* > 0.05 as indicated using two-tailed Mann–Whitney test

It is widely accepted that ischemia induces glutamate excitotoxicity and increased K^+^ levels in the extracellular space, which damage neurons and NG2-glial cells via necroptosis^11,27,28^. To explore whether ischemia affects Kir4.1 channels in NG2-glia, firstly we examined the basic membrane properties of both NG2-glia and astrocytes in a tMCAO mouse model (Fig. 2a). As illustrated in Fig. 2a^29^, the MCA of mouse was occluded for 30 min and then reperfused for 24 h before carrying out electrophysiological and immunohistochemical examinations. Hippocampal NG2-glia in ipsilateral SR showed depolarized RMPs and increased membrane resistances, as compared with that in the contralateral region (RMPs: contralateral, −84.5 ± 0.5 mV, *n* = 43 vs. ipsilateral, −79.7 ± 1.1 mV, *n* = 42, *P* < 0.0001, two-tailed unpaired *t*-test; membrane resistances: contralateral, 78 ± 4.2 MΩ, *n* = 43 vs. ipsilateral, 269.1 ± 29.4 MΩ, *n* = 42, *P* < 0.0001, two-tailed unpaired *t*-test, Fig. 2b–d). In contrast, neither RMPs nor membrane resistances in astrocytes displayed obvious changes in either side (RMPs: contralateral, −78.4 ± 0.9 mV, *n* = 22 vs. ipsilateral, −80.1 ± 1.1 mV, *n* = 16, *P* = 0.2238, two-tailed unpaired *t*-test; membrane resistances: contralateral, 17.3 ± 0.6 MΩ, *n* = 22 vs. ipsilateral, 18.2 ± 1.5 MΩ, *n* = 16, *P* = 0.5320, two-tailed unpaired *t*-test, Fig. 2b–d). In accord, an apparent reduction of macroscopic K^+^ currents in NG2-glia but not in astrocytes was observed in ipsilateral hippocampal infarction region of tMCAO mice (NG2-glia: contralateral, −936.96 ± 40.09 pA, *n* = 40 vs. ipsilateral, −257.98 ± 21.70 pA, *n* = 48 at holding voltage of −140 mV, *P* < 0.0001, two-tailed unpaired *t*-test; the slope conductances of macroscopic K^+^ currents in astrocytes: contralateral, 58.9 ± 2.4 nS, *n* = 21 vs. ipsilateral, 55.6 ± 4.6 nS, *n* = 15, *P* = 0.4907, two-tailed unpaired *t*-test, Fig. 3a–d).

Surprisingly, 100 µM Ba^2+^-induced Kir4.1 currents in NG2-glia exhibited 87% of the inward current loss at a holding voltage of −140 mV compared with that in the contralateral SR region in tMCAO mice (Kir4.1 currents in NG2-glia: contralateral, −530.9 ± 42.1 and 680.5 ± 68.5 pA, *n* = 22 vs. ipsilateral, −68.9 ± 17.3 and 368.8 ± 66.4 pA, *n* = 22 at holding voltages of −140 and +50 mV respectively, *P* < 0.0001 at −140 mV and *P* = 0.0022 at +50 mV, two-tailed unpaired *t*-test, Fig. 3a, b). In contrast, astrocytes in ipsilateral hippocampus of tMCAO mice did not show apparent reactive cell morphology nor any difference of Ba^2+^-sensitive Kir4.1 currents compared with that in the non-injured side^4,24^ (Kir4.1 currents in astrocytes: contralateral, −558.5 ± 66.0 and 1135.8 ± 137.8 pA, *n* = 19 vs. ipsilateral, −602.4 ± 82.8 and 1171.6 ± 224.5 pA, *n* = 14 at holding voltages of −140 and + 50 mV, respectively, *P* = 0.6745 at −140 mV and *P* = 0.8874 at +50 mV, two-tailed unpaired *t*-test, Fig. 3c, d). Although no apparent cell loss of total NG2 cell numbers nor reduction of NG2 protein expression level occurred in NG2-glia of infarcted hippocampus after tMCAO, the reactive morphology of NG2-glial cells was observed in the ipsilateral SR region, indicating an early response of NG2-glia, rather than astrocytes, to ischemia (total NG2-glia cell numbers: 203 ± 16 mm^−2^ in contralateral and 204 ± 13 mm^−2^ in ipsilateral, *P* > 0.05, Mann–Whitney test, *n* = 4 mice, Fig. 3e, f; total GFAP^+^ astrocyte cell numbers: 911 ± 112 mm^−2^ in contralateral and 840 ± 47 mm^−2^ in ipsilateral, *P* > 0.05, Mann–Whitney test, *n* = 4 mice, Fig. 3g, h, Supplementary Figure 3a, b).

To exclude possible ischemia-evoked rise of [H^+^]_i_ cations into the cell^23^, we loaded 50 mM HEPES in the intracellular solution of the patch pipette to buffer [H^+^]_i_ in NG2-glia and found it had no effect on the loss of inward K^+^ currents in infarcted hippocampal region of tMCAO mice (total inward K^+^ currents at holding voltage of −140 mV: contralateral, −940.8 ± 102.5 pA, *n* = 5 vs. ipsilateral, −232.9 ± 51.4 pA, *n* = 9, *P* < 0.0001, two-tailed unpaired *t*-test, Supplementary Figure 4a, b). Interestingly, bath application of acidic artificial cerebrospinal fluid (ACSF) at pH 6.0 induced a decrease of macroscopic K^+^ currents in NG2-glia (total inward K^+^ currents at holding voltage of −140 mV: −751.1 ± 84.7 pA in pH 7.4 ACSF vs. −379.0 ± 72.2 pA in pH 6.0, *n* = 10, *P* = 0.0027, two-tailed paired *t*-test, Supplementary Figure 4c, d), suggesting an extrinsic acidic mechanism in tMCAO participated in the Kir current inhibition in NG2-glia^30,31^.

### Pathological role of Kir4.1 channels following ischemia

**Fig. 4:**
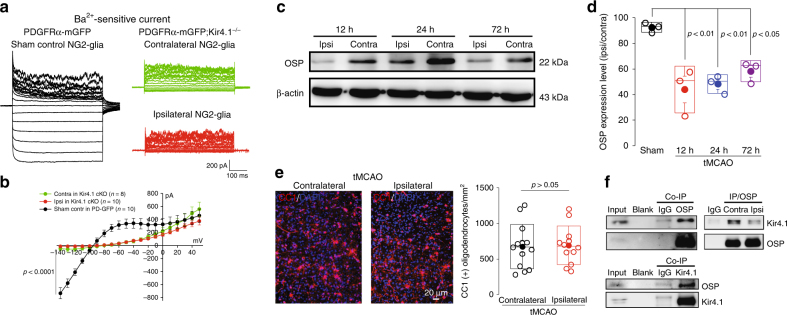
Kir4.1 channel deficiency in NG2-glia causes a decrease of OSP expression in tMCAO. **a** Representative traces show Ba^2+^-sensitive currents in both contralateral (in green) and ipsilateral (in red) NG2-glia in PDGFRα-mGFP; Kir4.1^−/−^ transgenic mouse after 30 min tMCAO at postnatal 8 weeks. The Kir4.1 currents in ipsilateral hippocampal NG2-glia show an apparent reduction compared with its sham control (in black) in PDGFRα-mGFP mice. However, there is no further decrease of Kir4.1 currents in ipsilateral NG2-glia compared with contralateral NG2-glia. **b** Average *I/V* plot is for Ba^2+^-sensitive currents in NG2-glia in tMCAO from Kir4.1 cKO and the sham control in PDGFRα-mGFP mice. The error bars represent s.e.m. *n* indicates the cell numbers recorded. Statistical significance was assessed as indicated using two-tailed unpaired *t*-test. **c** Representative Western blots show OSP expression levels in both contralateral and ipsilateral regions after 30 min tMCAO mice at postnatal 8 weeks with 12, 24, and 72 h reperfusion respectively. **d** Bar graph shows 44%, 48%, and 58% decrease of total OSP protein levels in ipsilateral regions after tMCAO at different time checking points compared with the sham control. *n* represents the number of mice. Statistical significance was assessed as indicated using ANOVA followed by Dunnett Multiple Comparison tests with sham as control. **e** Representative images of CC1+ oligodendrocytes in both contralateral and ipsilateral cortex after tMCAO mice at postnatal 8 weeks. Scale bar: 20 µm. The bar graph on the right indicates that there was no difference of oligodendrocyte numbers between the infarction and contralateral region of tMCAO. The error bars represent s.e.m. *n* = 5 mice. *P* > 0.05 as indicated using two-tailed Mann–Whitney test. **f** The co-Immunoprecipitation results on the left panels show OSP reciprocally binding with Kir4.1 in WT mouse brain tissue. The panel on the right shows the reduction of Kir4.1 and OSP interactions in ipsilateral brain tissue of tMCAO mice at postnatal 8 weeks compared with its contralateral side when the same quantity of OSP protein is immunoprecipitated in these two lysates

**Fig. 5:**
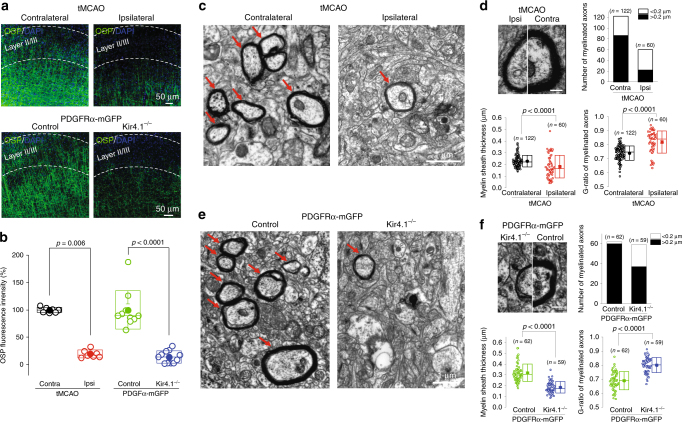
Kir4.1 channel deficiency in NG2-glia contributes to the myelin loss in axons after tMCAO. **a** Representative images of OSP immunofluorescence in 30 min tMCAO mice at postnatal 8 weeks after 24 h reperfusion (upper panel) and in Kir4.1 cKO mice at postnatal 4 weeks (lower panel). Note the pale and dim OSP staining in cortical layer II/III in ipsilateral side compared with that in contralateral side. Similar reduction of OSP fluorescence intensity was seen in Kir4.1 cKO mice compared with its control. Scale bars: 50 µm. **b** The graph summary shows the percentage of OSP reduction in these two groups. The error bars represent s.e.m. *n* = 3 mice per group. Statistical significance was assessed as indicated using two-tailed Mann–Whitney test. **c** Electron micrographs demonstrate the presence of impaired axons with demyelination in ipsilateral cortex compared with its contralateral region after 30–45 min tMCAO mice at postnatal 8 weeks. In contralateral cortex of tMCAO, axons show normal myelin which exhibits dark, ring-shaped sheaths surrounding the axon, as indicated by arrowheads. Scale bar: 1 µm. **d** The magnified EM image shows a comparison of one myelin sheath surrounding axon in ipsilateral with its contralateral side after tMCAO. Scale bar: 1 µm. Bar graph and box plots represent average myelinated axon numbers, myelin sheath thickness, and *G*-ratio between ipsilateral cortex after tMCAO with its contralateral side. The data were normally distributed and statistical significance was assessed using two-tailed unpaired *t*-test, *P-*values are indicated, *n* represents analyzed axons from four mice. **e** Electron micrographs show the presence of impaired axons with demyelination in Kir4.1 cKO mice at postnatal 4 weeks compared with control mice. Scale bar: 1 µm. **f** The magnified EM image shows a comparison of one myelin sheath surrounding axon in Kir4.1 cKO mouse with its control. Scale bar: 1 µm. Bar graph and box plots represent average myelinated axon numbers, myelin sheath thickness, and *G*-ratio between Kir4.1-deficient mice with their control. The data were normally distributed and statistical significance was assessed using two-tailed unpaired *t*-test, *P-*values are indicated, *n* represents analyzed axons from three mice per group

To directly illustrate the pathological role of Kir4.1 channels in NG2-glia after ischemia, we first obtained electrophysiological recordings from PDGFRαCre^ER^-mGFP; Kir4.1 ^f/f^ transgenic mice after tMCAO. Whole-cell patch recordings from Kir4.1-deficient NG2-glia further confirmed that there was no additional reduction of Ba^2+^-sensitive Kir4.1 currents in the ipsilateral hippocampus compared with its contralateral side in cKO mice (Ba^2+^-sensitive currents in Kir4.1 cKO NG2-glia: contralateral, −56.4 ± 36.5 pA, *n* = 8 vs. ipsilateral, −13.5 ± 4.6 pA, *n* = 10 at holding voltages of −140 mV, *P* = 0.2084, two-tailed unpaired *t*-test, Fig. 4a, b), suggesting a major impairment of Kir4.1 channels in NG2-glia from tMCAO mice. Previous studies have suggested that hypoxia and ischemia mainly impair NG2-glial cells’ self-renewal capacity, which therefore causes functional loss of maturation, differentiation, and myelination in oligodendrocytes^2^^,^^3,22,28^. However, our results showed an impact of Kir4.1 in NG2-glia on demyelination in ischemic stroke as evidenced by the facts that: (a) the oligodendrocyte-specific protein (OSP/Claudin-11) exhibited a continuous loss in ipsilateral as compared with its contralateral side (OSP reduction rate in 12, 24, and 72 h reperfusion of tMCAO are 44.02 ± 10.55% (*P* < 0.01), 48.3 ± 4.11% (*P* < 0.01), and 58.3 ± 4.78 % (*P* < 0.05) compared with its sham control 92.62 ± 2.24%, respectively, Fig. 4c, d), which was not due to the change in oligodendrocytes, as CC1-labeled oligodendrocytes number did not show distinct reduction in the infarction region of tMCAO mice (CC1^+^ cell numbers, contralateral, 676 ± 87 mm^−2^ vs. ipsilateral, 692 ± 78 mm^−2^, *n* = 5 mice, *P* > 0.05, Mann–Whitney test, Fig. 4e)^32^; (b) the Co-Immunoprecipitation (Co-IP) results indicated that OSP protein can reciprocally associate with Kir4.1 channel protein and this binding interaction was reduced in the infarction tissue compared with its contralateral region of tMCAO mice (Fig. 4f); (c) a significant loss of OSP (Claudin-11) immunofluorescence intensity occurred in infarction cortex of tMCAO mice and in Kir4.1 cKO mice (OSP fluorescence intensity in tMCAO mice: contralateral, 99.99 ± 1.84% vs. ipsilateral, 19.77 ± 2.71%, *P* = 0.006, Mann–Whitney test; OSP fluorescence intensity in PDGFRα-mGFP; Kir4.1^−/−^: control, 99.99 ± 11.02% vs. Kir4.1^−/−^, 14.25 ± 3.38%, *P* < 0.0001, Mann–Whitney test, *n* = 3 mice per group, Fig. 5a, b, Supplementary Figure 5), which suggested a strong correlation between NG2-glia expressing Kir4.1 and myelin formation; (d) Electron microscopy results further demonstrated remarkable morphological changes of myelin in axons in both ischemic mice and in Kir4.1-deficient mice, as the thickness of myelin sheaths and *G*-ratio of myelinated axons are both impaired in ipsilateral side of tMCAO mice and in Kir4.1 cKO mice (myelin sheath thickness after tMCAO: contralateral, 0.2300 ± 0.0045 µm vs. ipsilateral, 0.1817 ± 0.0126 µm, *P* < 0.0001, two-tailed unpaired *t*-test; *G*-ratio of myelinated axons after tMCAO: contralateral, 0.7403 ± 0.0047 vs. ipsilateral, 0.8192 ± 0.0102, *P* < 0.0001, *n* = 122 and 60 axons from 4 mice, two-tailed unpaired *t*-test; myelin sheath thickness in PDGFRα-mGFP; Kir4.1^−/−^: control, 0.3188 ± 0.0102 µm vs. Kir4.1^−/−^, 0.1844 ± 0.0069 µm, *P* < 0.0001, two-tailed unpaired *t*-test; G-ratio of myelinated axons in PDGFRα-mGFP; Kir4.1^−/−^: control, 0.6892 ± 0.0082 vs. Kir4.1^−/−^, 0.7999 ± 0.0071, *P* < 0.0001, *n* = 62 and 59 axons from three mice per group, two-tailed unpaired *t*-test, Fig. 5c–f).

## Discussion

In summary, our evidence strongly demonstrates that (1) NG2-glia express Kir4.1 (*kcnj10*) during early development and adult life, as shown by RNA-sequencing, single-cell RT-PCR, as well as electrophysiology in a conditional Kir4.1 knock-out mouse; (2) Kir4.1 channel currents constitute a large portion of total inward K^+^ channel currents in NG2-glia and an initial deficit of Kir4.1 channels in NG2-glia but not astrocytes occurred in a tMCAO mouse model; (3) deficits of Kir4.1 channels in NG2-glia contribute to the myelin loss of axons in tMCAO which is potentially through the association of Olig-specific protein (OSP/Claudin-11). Our data systematically showed that NG2-glia functionally express Kir4.1 in adult brain and the impairment of myelin in axons was possibly caused by the deficits of Kir4.1 in NG2-glia after ischemia. NG2-glia, known as OPCs, sustain oligodendrocyte maturation, differentiation, and myelination during early brain development^2,13,14,16^. It has been reported that the K^+^ channel family plays an important role in axon’s myelination as well as in myelin-related brain damage^33–36^. Our data also support that K^+^ channel subtype Kir4.1 which is expressed in NG2-glia is crucial to the myelin formation during brain development and could contribute to the myelin loss after ischemia (Fig. 5). In normal brain, Kir4.1 upregulates in NG2-glia to sense local K^+^ rises induced by neuronal activities^10^. However, in conditions of brain damage such as ischemia, both elevated [K^+^]_o_ and low pH exacerbate Kir4.1 channel openings in NG2-glia^30,31^, which in turn impairs OSP/Claudin-11 to cause a direct loss of myelin in axons, although an intrinsic mechanism of Kir4.1-OSP interaction remains unclear^35^. By providing a close spatial contact and synaptic interactions with surrounding neurons^10,12,15^, NG2-glia enable a fast response during normal healthy conditions and could exhibit higher vulnerability than other types of glial cells as well when triggered by hypoxia-induced ischemic signals. Therefore, NG2-glia not only play a role as cell reservoir for sustaining oligodendrocyte maturation and differentiation in the brain, they could also respond rapidly by directly interacting with neurons in myelination during the ischemic disease process. The observation that deficits of Kir4.1 channels in NG2-glia contribute to the loss of myelin in tMCAO highlights a new role of NG2-glia in physiological/pathological conditions in the brain and sheds light on a potential therapeutic target of NG2-expressing Kir4.1 channels for the treatment of ischemic stroke.

## Methods

### Animals

All animal procedures complied with the animal care standards set forth by the US National Institutes of Health and were approved by the Institutional Animal Care and Use Committee (IACUC), Shanghai Jiao Tong University School of Medicine. Only male mice were used. Mice were kept on a C57BL/6 background and under a 12 h–12 h light–dark cycle with food and water provided ad libitum from the cage lid. NG2DsRedBAC (Cspg4-DsRed.T1; JAX strain 008241| NG2DsRed), PDGFRα-creERT (JAX strain 018280| B6N.Cg-Tg (Pdgfra-cre/ERT)467Dbe/J), Rosa26-mGFP (JAX strain 007676 mT/mG| B6.129(Cg)-Gt(ROSA)26Sor^tm4(ACTB-tdTomato,-EGFP)Luo^/J), Kir4.1^f/f^ (JAX strain 026826| B6.129-Kcnj10^tm1Kdmc^) were obtained from The Jackson Laboratory (USA). To induce Cre recombinase in PDGFRα-creERT; Kir4.1^f/f^ mice, 120 mg kg^−1^ tamoxifen (ABCONE, T56488-5G) dissolved in sunflower seed oil (SIGMA-ALDRICH) was intraperitoneally injected for five consecutive days starting from the postnatal day 10. To induce Cre recombinase in PDGFRα-creERT; Rosa26-mGFP mice, 120 mg kg^−1^ tamoxifen^37^ was intraperitoneally injected for 10 consecutive days between postnatal day 45 and 60.

### MCAO model in mice

The brain ischemic stroke mouse model was established as described before with slight modifications^38^. In brief, six- to eight-week-old male mice (~22 g) were anesthetized with an injection of 5% chloral hydrate (20 mL kg^−1^). Rectal and temporalis muscle temperature was maintained at 37 ± 0.5 °C with a thermostatically controlled heating pad and lamp. A suture of 0.105 mm in diameter with a 0.2-mm-diameter tip (Jia-Ling Biological Technology, China) was inserted into the internal carotid artery (ICA) through a cut of the external carotid artery to occlude the MCA for 30 min. Regional cerebral blood flow was monitored by laser Doppler flowmetry (VMS-LDF2; Moor Instruments Ltd, UK). Mice showing less than 20% reduction in cerebral blood flow at the core regions of the MCA territory were excluded from the study. After reperfusion for 24 h, animals were sacrificed for experimentation and the infarction volumes of the brain were determined by TTC (BBI Life Sciences, A610558-0025) staining.

### Preparation of brain slices and electrophysiological recordings

For preparation of brain slices, mice were deeply anesthetized with 5% chloral hydrate and intracardially perfused with ice-cold carbogenated (95% O_2_, 5% CO_2_) ACSF containing: 125 mM NaCl, 2.5 mM KCl, 1 mM MgCl_2_, 2 mM CaCl_2_, 1.25 mM NaH_2_PO_4_, 25 mM NaHCO_3_, and 12.5 mM d-glucose. Coronal sections of the brain were cut into 300 µm thickness (VT1200S; Leica Microsystems, Germany) and allowed to equilibrate for at least 1 h at 31 ˚C in ACSF which continuously bubbled with a mixture of 95% O_2_/5% CO_2_ gas. Intracellular solutions for NG2-glial and astrocytic recordings consisted of (in mM) the following: 125 K-gluconate, 15 KCl, 8 NaCl, 10 HEPES, 0.2 EGTA, 3 Na_2_-ATP, and 0.3 Na-GTP and pH set to 7.3 (~305 mOsm). Slices were visualized with an upright epifluorescent microscope (BX51WI; Olympus, Tokyo, Japan) equipped with differential interference contrast optics and an infrared CCD camera (optiMOS, Q IMAGING; Olympus, Tokyo, Japan). Whole-cell recordings were made from NG2-glia and astrocytes in hippocampal CA1 stratum radiatum, with a MultiClamp 700B amplifier (Molecular Devices, Sunnyvale, CA, USA). Signals were low-pass filtered at 2 kHz and sampled at 20 kHz using Digidata 1550A (Molecular Devices) in all experiments.

### Immunohistochemistry and image analysis

Mice were anesthetized and perfused through the ascending aorta with a solution of normal saline for ~3 min, followed by 2% paraformaldehyde in 0.1 M phosphate buffer for 5 min. Brains were removed and post-fixed in 2% paraformaldehyde at 4 °C overnight and then cut into 40-µm-thick coronal sections including cortex and hippocampus. Slices were incubated in permeable buffer (0.3% Triton X-100 in phosphate-buffered saline (PBS)) for 15 min and then blocked with donkey serum (Ruite Biotechnology, w9030-05) (5% in PBS-T: PBS with 0.1% Triton X-100) for 2 h at room temperature. The primary antibodies used include rabbit antibody to NG2 (1:300; Millipore), chicken antibody to GFAP (1:500; Abcam), mouse antibody to CC1 (1:500; Millipore), rabbit antibody to OSP (1:300; Abcam), chicken antibody to GFP (1:500; Abcam). The corresponding secondary antibodies include Goat anti-Rabbit IgG 647 (Cell Signaling, 4414S, USA), Goat anti-Mouse IgG 647 (Cell Signaling, 4401S, USA), Goat anti-Rabbit IgG 488 (Cell Signaling, 4412S, USA). Sections were incubated with 4′,6′-diamidino-2-phenylindole dihydrochloride (DAPI, 1:1000; Cell Signaling) for 15 min to label nuclei at room temperature and mounted on glass slides in Fluoromount™ Aqueous Mounting Medium (AQUA-MOUNT, REF 13800). All slice images were acquired on a Leica TCS SP8 confocal microscope with HC PL APO CS2 ×20/0.75 DRY and/or HC PL APO CS2 ×40/1.30 OIL objective. Images analysis was performed by Image-Pro Plus (Media Cybernetics). The images of different channel were thresholded; cell numbers were determined according the DAPI channel threshold image. For each mouse, at least three slices were counted and averaged as one sample.

### Western blots and Co-IP

For evaluation of protein expression by Western blot analysis, mice were anesthetized with 5% chloral hydrate (20 mL kg^−1^) and brains rapidly removed and placed into ice-cold PBS. Fresh tissues including hippocampus were rapidly dissected bilaterally in ice-cold PBS under ×10 magnification (Zeiss) and tissue samples were rapidly frozen on dry ice. Total protein was extracted from individual tissue samples using a lysis buffer containing: 50 mM Tris HCl, 150 mM NaCl, 1% NP-40, 0.5% deoxycholate, 0.1% SDS, protease inhibitors cocktail (Cat. No. 04 693 132001; Roche), pH 7.6 and centrifuged at 12,000 *g* for 20 min at 4 °C. The lysates were centrifuged to remove the insoluble deposit and the supernatants were measured with a BCA kit (Thermo Fisher Scientific, USA). The supernatants were denatured at 65 ˚C for 30 min and then the proteins were separated on a Tris-glycine gel and transferred into a polyvinlyidene difluoride membranes (BIO-RAD #162-0177), which were blocked for 2 h in TBS-T (TBS with 0.1% Tween 20, pH 7.6) containing 3% bovine serum albumin (BSA) (BBI Life Sciences, A600332-0100) and incubated with primary antibody at 4 °C overnight. The primary antibodies used for Western blotting were rabbit anti-NG2 (1:300; Millipore), chicken anti-GFAP (1:500; Abcam), rabbit anti-Kir4.1 (1:500; Alomone lab), rabbit anti-OSP (1:250; Abcam). The membranes were subsequently washed with TBS-T and incubated for 2 h with HRP-conjugated anti-rabbit or anti-mouse immunoglobulin G (1:5000) at room temperature. Western blots were visualized using Clarity™ Western ECL Substrate (Bio-Rad, Hercules, USA) and exposed to the Tanon Chemiluminescence imaging system. Equivalence of protein loading was corrected by probing for β-actin or GAPDH. For quantification, the optical density of the gel bands was determined using ImageJ Software v1.30 (US National Institutes of Health). Immunoprecipitations were performed using the Pierce Classic IP kit (Thermo Scientific Pierce, USA) according to the manufacturer’s instructions. For co-immunoprecipitation, extracts from isolated brain tissues were precleared with Protein G-agarose at 4 °C for 30 min. Then 4 µg of desired antibodies or control normal IgG was added to the lysates and incubated with protein G-agarose beads overnight at 4 °C. The next day, the beads were washed with the IP lysis/Wash buffer three times. The immunoprecipitants were eluted using 2× Non-reducing Lane Marker Sample Buffer. All the immunoprecipitated samples were subjected to SDS Western blotting.

### RNA and protein extraction from NG2-glia by FACS

Mice expressing DsRed under NG2-glia-specific CSPG4 promoter (NG2DsRedBAC) were used to purify NG2-glia by FACS. The brain tissues from NG2DsRed mice at P14 were dissociated following published guidelines^39^ with slight modifications. Briefly, the brain tissues were dissected and digested for 90 min at 36˚C in 50 mL centrifuge tubes with 10 ml papain solution (1× EBSS, 0.46% glucose, 26 mM NaHCO_3_, 50 mM EDTA, 75 U mL^−1^ DNase I, 300 units of papain, 2 mM l-cysteine) bubbling with 5% CO_2_, 95% O_2_. After digestion, the tissue was washed four times with ovomucoid solution (1× EBSS, 0.46% d-glucose, 26 mM NaHCO_3_, 1 mg mL^−1^ ovomucoid, 1 mg mL^−1^ BSA, and 60 U mL^−1^ DNase I) and mechanically dissociated with two fire-polished borosilicate glass pipettes with different bore sizes. A bottom layer of concentrated ovomucoid solution (1× EBSS, 0.46% d-glucose, 26 mM NaHCO_3_, 1 mg mL^−1^ ovomucoid, 1 mg mL^−1^ BSA, and 60 U mL^−1^ DNase I) was added to the cell suspension. The tubes were centrifuged at room temperature at 300 *g* for 10 min, and the resultant pellet was re-suspended in D-PBS with 0.02 % BSA and 13 U mL^−1^ of DNase I, and filtered with a 40 µm mesh. FACS was performed in a BD FACSAria II Flow Cytomerter (BD Bioscience) with a 70 µm nozzle using standard methods at Shanghai Jiao Tong University, Core Facility of Basic Medical Sciences and analyzed with FlowJo software. For RNA extraction, sorted cells were collected in D-PBS with 0.1% BSA, and centrifuged for 10 min a`t 4 °C and 2000 *g*. The RNA was extracted from the pelleted cells using Trizol reagent (Thermo Scientific, Pierce, USA). For protein extraction, cells were collected in D-PBS and, right after FACS, cells were incubated with lysis buffer (150 mM NaCl, 1% Triton X-100, 12 mM Na^+^-deoxycholate, 3.5 mM sodium dodecyl sulfate, 50 mM Tris pH 8, and Protease Inhibitor cocktail) at 4 °C for 40 min.

### Single-cell RT-PCR and RNA-sequencing

Single NG2-glia with GFP fluorescence labeling from PDGFRαCreER; mGFP mice at postnatal 7 weeks was selected and aspirated into a glass pipette from hippocampal acute slices following a method described previously with slight changes^40^. In brief, cells were picked promptly by micromanipulation and immediately placed in lysis buffer. To minimize the changes in gene expression and meet the quality requirement for cDNA used to construct sequencing libraries, all NG2-glial cells were collected within 3 h after slice preparation. The selected NG2-glia were processed for single-cell RNA extraction and reverse transcription within 1 h and were subjected to cDNA amplification and purification. Single-cell cDNA was amplified using KAPA HiFi HotStart ReadyMix (2×; KAPA Biosystems, Cat. No. KK2601) according to the manufacturer’s protocol. The RNA probes were generated using the following primers: NG2, Forward primer: GTTGGGATGCTTGCTGGTAT; Reverse primer: TGAAAGCTGCAGAAGCAGAA; Kir4.1, Forward primer: CTGCCCCGCGATTTATCAGA; Reverse primer: CATTCTCACATTGCTCCGGC. GAPDH, Forward primer: GGCAAATTCAACGGCACAGT; Reverse primer: TAGGGCCTCTCTTGCTCAGT.

For RNA-seq transcriptome experiment, PDGFRαCreER; mGFP mouse was anesthetized by isoflurane and the brain was removed. The fresh brain tissue was cut into small pieces and the minced tissue was incubated in 15 unit mL^−1^ papain at 31 °C for 45 min. The digestion was stopped by protease inhibitor solution (Ovomucoid)^41^. After which, the tissue was immediately triturated and the isolated cells were seeded on coverslips. Single GFP-labeled NG2-glia was selected and aspirated into a glass pipette. The total RNA of NG2-glia in lysis buffer was converted to cDNA using the Smart-seq2 protocol and the cDNA was preamplified as described previously^40,42^. Illumina libraries were prepared using the commercially Sample Preparation kit (Nextera XT DNA Library Prep Kit) according to the manufacturer’s instructions. The barcoded single-cell Illumina libraries of each experiment were pooled and sequenced for 2 × 75-base Paired-End reads on Illumina NextSeq500 sequencing system at the Sequencing Core of Shanghai Institute of Immunology, Shanghai Jiao Tong University School of Medicine. Sequencing reads were inspected by Fastqc 0.11.3 to check the reads quality and then aligned to the GRCm38/mm10 assembly of the mouse genome using Tophat 2.1.0 with the default options. FPKM (fragments per kilobase of exon per million fragments) values of each gene were obtained by Cufflinks 2.2.1 using genome annotation from UCSC (University of California, Santa Cruz). The GTF (gene transfer format) file was modified to update the genes encoding all inwardly rectifying potassium channel family members to the latest version archived in NCBI. To compare the expression level across different samples, FPKM values were transformed into TPM (transcripts per million) values after exclusion of microRNAs, small nucleolar RNAs, and rRNAs as previously reported^43^. The TPM values of all inwardly rectifying K^+^ channel family members (Supplementary Table 1) were plotted as heat map as shown in Fig. 1c.

### Electron microscopy

The procedure was conducted as described before with slight changes^44^. In brief, sections were rinsed in phosphate buffer and immersed in a solution of 1% osmium tetroxide in phosphate buffer for 1 h, then rinsed in phosphate buffer and gradually dehydrated on a series of ethanol from 30% to 70%. After that, the sections were stained with a solution of 1% uranyl acetate in 70% ethanol for 1 h and further dehydrated in ethanol. After dehydration was completed the sections were cleared in propylene oxide and infiltrated with Epon resin overnight at room temperature. The following day the sections were flat-embedded in new Epon resin and allowed to polymerize in an oven at 60 °C for 72 h. Ultrathin sections (90 nm thick) were obtained using a Leica EM UC6 ultramicrotome (Leica Microsystems, Wetzlar, Germany), observed and photographed using a Hitachi TEM model H-7650 (Hitachi, Japan) equipped with an AMT digital camera (Danvers, MA). Selection of regions of interest was performed on a Nikon Eclipse 50i light microscope, carefully identifying anatomical regions and re-dissecting these regions for ultramicrotomy. The *G*-ratio for single labeled axons (longitudinally or transversally cut) was calculated from calibrated electron microscopy images as the diameter of the axon divided by the total diameter of the axon including the myelin sheath using ImageJ.

### Data analysis

All statistical tests were run in GraphPad InStat 3. The graphs were created in Origin 8 and assembled in CorelDraw 12. Data are presented as mean ± s.e.m. For each set of data to be compared, we determined in GraphPad Instat whether the data were normally distributed or not. If they were normally distributed we used parametric tests, as listed in the text. If the data were not normally distributed we used non-parametric tests, as indicated in the text. Paired and unpaired Student’s two-tailed *t-*tests (as appropriate and as indicated in the text) and two-tailed Mann–Whitney tests were used for most statistical analyses. For electrophysiological experiments, *n* values represent the number of recorded cells. For all biochemistry and immunohistochemistry experiments, *n* values represent the number of mice. Statistical significance was set at *P < 0.05, **P < 0.01, ***P < 0.0001, n.s., not significant.

### Chemicals and drug application

All chemicals for electrophysiology were purchased from Sigma-Aldrich. The potassium blocker barium chloride was dissolved in double-distilled water at 50 mM and stored in aliquots at −20 °C. The blocker was bath applied at 100 µM working concentration.

### Data availability

The authors declare that all data supporting the findings of this study are available within the article and its supplementary information files. All relevant data not present within the manuscript or supplementary files are available from the corresponding author upon reasonable request. RNA-sequencing data are deposited at the Sequence Read Archive with SRA accession number SRP146737.

## Electronic supplementary material

Supplementary information
