# Supplementary material for: Kir4.1 channels in NG2-glia play a role in development, potassium signaling, and ischemia-related myelin loss
Source: Commun Biol. 2018 Jun 28;1:80. doi: 10.1038/s42003-018-0083-x (PMC6123808; doi:10.1038/s42003-018-0083-x)
Supplement: Supplementary file 1 — Supplementary information [file 42003_2018_83_MOESM1_ESM.pdf]

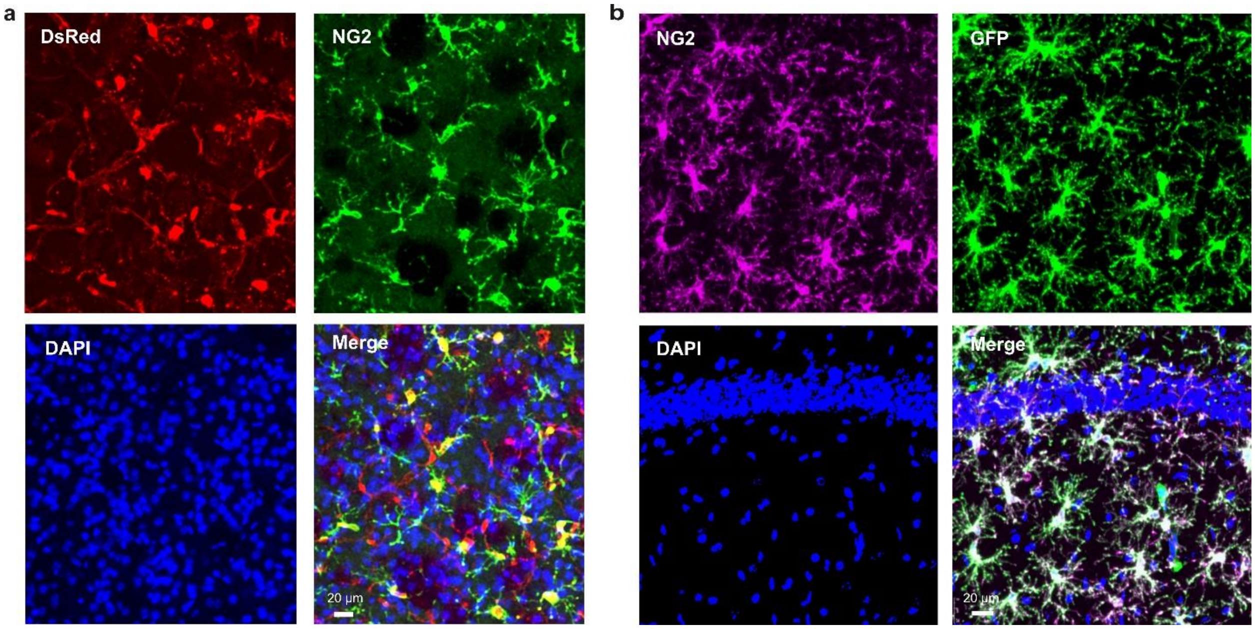

Supplementary Figure 1. NG2-glia are widely distributed in the transgenic mouse brain. a Representative images show the colocalization of NG2 antibody labeled NG2-glia (in green) with RFP labeled cells (in red) in the striatum of NG2DsRedBAC transgenic mouse at postnatal 2 weeks. The percentage of colocalization was 66 ± 4 % (n = 3 mice). **b** Representative images show the colocalization of NG2 antibody labeled NG2-glia (in magenta) with GFP positive cells (in green) in the hippocampus of PDGFRα-CreER; Rosa26-mGFP transgenic mouse brain at postnatal 8-10 weeks. The PDGFRα-CreER; Rosa26-mGFP mice show an intact NG2-glia morphology and a high colocalization rate with NG2+ cells in adult brain. The percentage of colocalization was 99 ± 1 % (n = 4 mice). Scale bars: 20 μm.

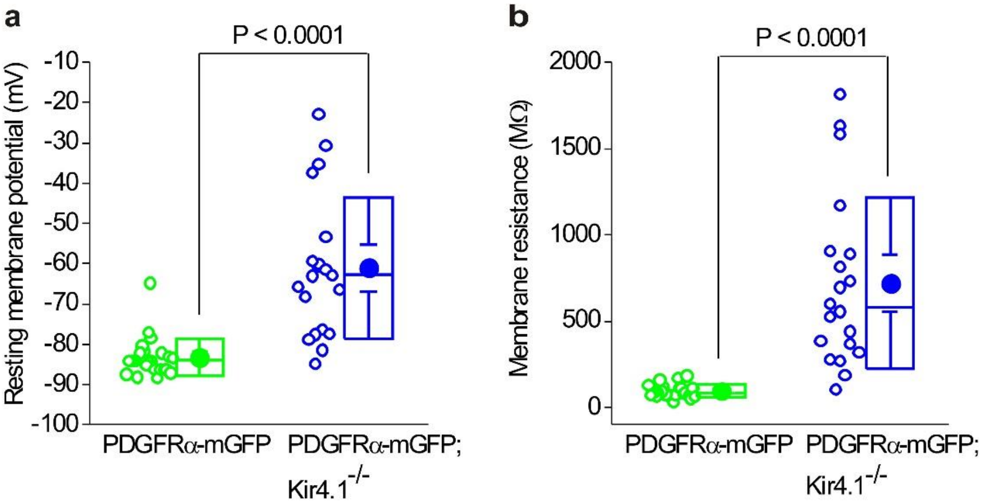

Supplementary Figure 2. Membrane properties of NG2-glia in wild type mice and Kir4.1 cKO mice. a Box plot shows the resting membrane potentials (RMPs) for NG2-glia in both PDGFRα-mGFP and PDGFRα-mGFP; Kir4.1$^{-/-}$ transgenic mice at postnatal 8 weeks. **b** Box plot shows the membrane resistances between −70 and −60 mV for NG2-glia in both PDGFRα-mGFP and PDGFRα-mGFP; Kir4.1$^{-/-}$ transgenic mice at postnatal 8 weeks. Note that NG2-glia with Kir4.1 deletions in Kir4.1 cKO mice show overall a distinct depolarization of RMP and increased membrane resistance. n = 25 and 20 cells from each group. The data are presented as mean ± s.e.m. The data were normally distributed and statistical significance was assessed using two-tailed unpaired t-test, P values were indicated.

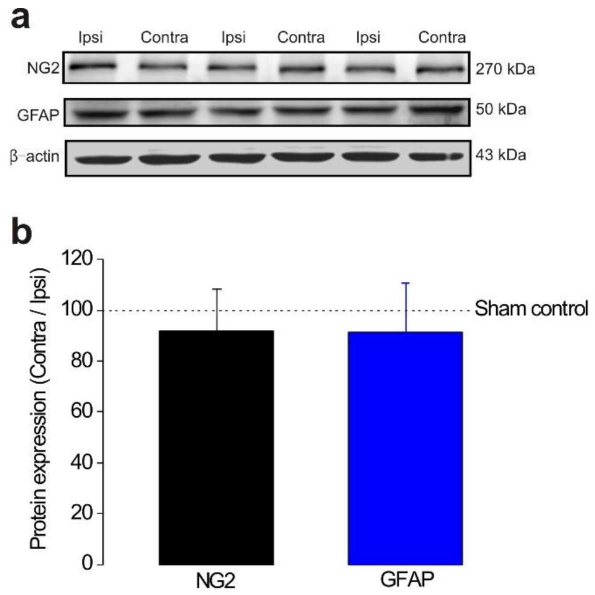

Supplementary Figure 3. NG2 and GFAP protein expressions from contralateral and ipsilateral brain tissues of tMCAO mice. a Representative Western blots for NG2 and GFAP expressions in both contralateral and ipsilateral hippocampus after 30 min tMCAO mice with 24 h reperfusion respectively at postnatal 8 weeks. **b** Averaged bar graph shows no obvious change of NG2 and GFAP protein in both sides of the brain after 30 min tMCAO compared with that in sham control. Error bars represent s.e.m. n = 3 mice. $P > 0.05$. The data were not normally distributed and statistical significance was assessed using two-tailed Mann-Whitney test.

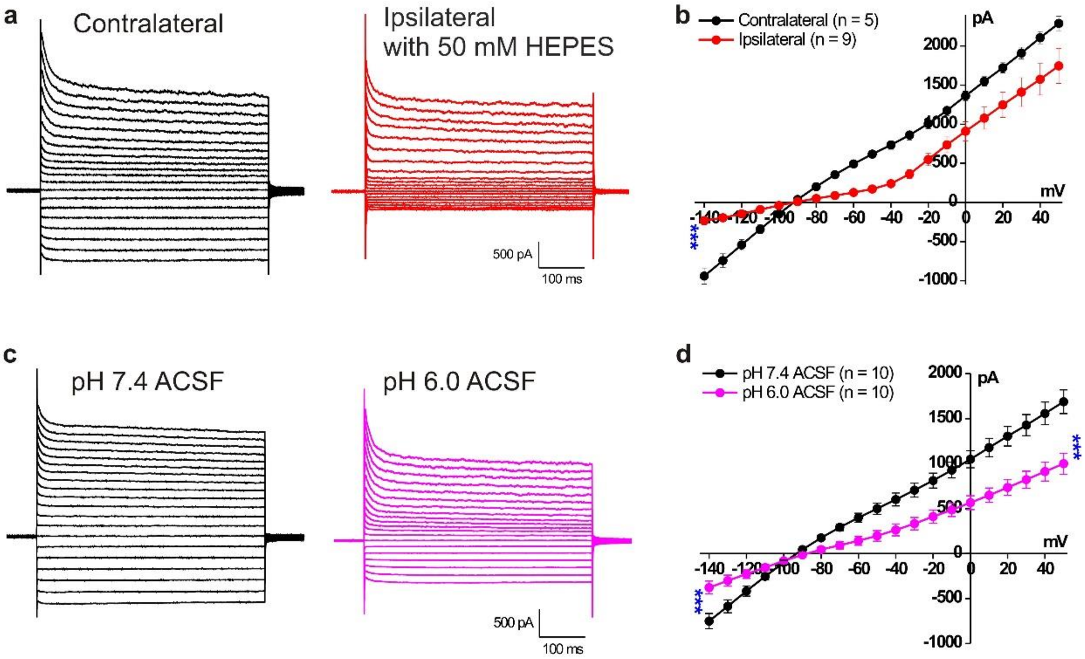

Supplementary Figure 4. Extracellular pH change reduces macroscopic K^+ currents in NG2-glia. a Representative traces show total macroscopic currents of NG2-glia in hippocampal SR region of the mouse at postnatal 8 weeks evoked by a series of voltage steps in whole-cell patch clamp mode (between -140 mV and +50 mV, 10 mV increments). Compared to the contralateral hippocampal NG2-glia in tMCAO mice at postnatal 8 weeks, addition of 50 mM HEPES to buffer $[H^+]_i$ had no effect on the reduction of total K^+ currents in infarction ipsilateral NG2-glia. **b** Average *I/V* plots are illustrated and error bars represent s.e.m. Statistical significance was assessed using two-tailed unpaired t-test. ***, $p < 0.0001$. **c, d** Representative traces (**c**) show total macroscopic currents of NG2-glia in pH 7.4 normal ACSF (black traces) and after application of pH 6.0 acidic ACSF (magenta traces). Both inward and outward K^+ currents were inhibited significantly as shown in summary average *I/V* plots in (**d**). Statistical significance was assessed using two-tailed paired t-test. ***, $p < 0.0001$.

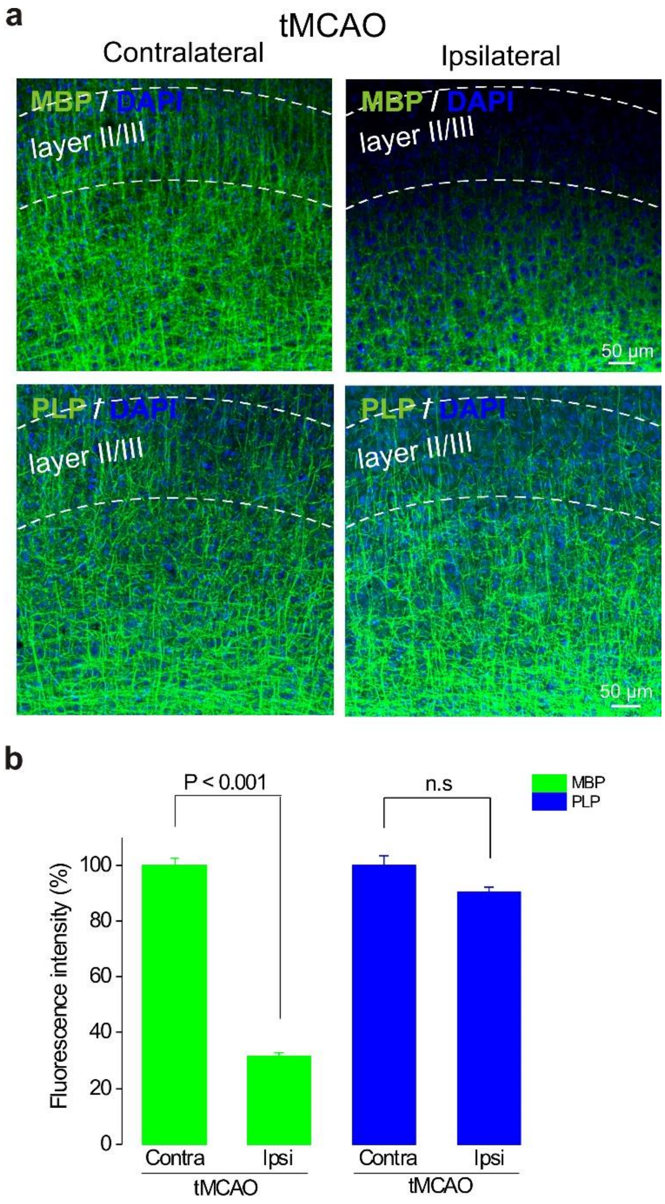

Supplementary Figure 5. MBP and PLP immunofluorescence after 30 min tMCAO mice at postnatal 8 weeks. a Representative images of MBP and PLP immunofluorescence after 30 min tMCAO mice at postnatal 8 weeks. Note MBP immunofluorescence intensity had a reduction while there is no obvious change of PLP labeling in ipsilateral cortical layers compared with that in its contralateral side. Scale bars: 50 µm. **b** Average fluorescent intensity as shown in a bar graph summary. The error bars represent s.e.m. n = 4 and 3 mice for each group. Statistical significance was assessed using two-tailed Mann-Whitney test. P values were indicated.

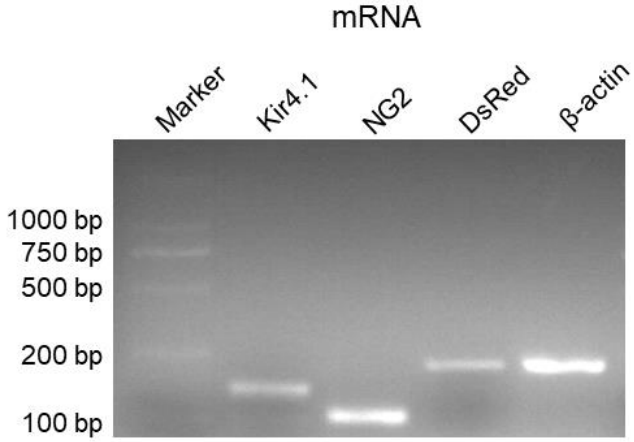

Supplementary Figure 6. Full-sized scan of PCR for Kir4.1 mRNA expression in purified NG2-glia from NG2DsRed BAC transgenic mouse brain at postnatal 2 weeks in Figure 1b.

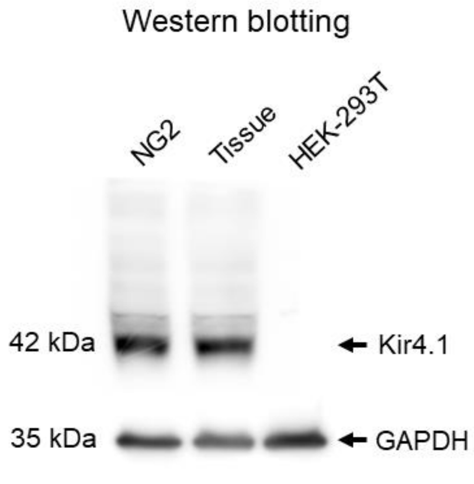

Supplementary Figure 7. Full-sized scan of Western blotting for Kir4.1 expression in purified NG2-glia from NG2DsRed BAC transgenic mouse brain at postnatal 2 weeks in Figure 1b.

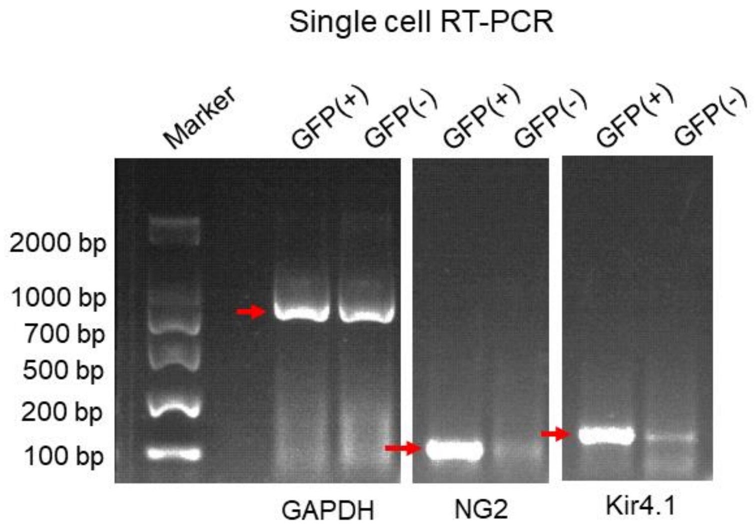

Supplementary Figure 8. Full-sized scan of single cell RT-PCR for Kir4.1 mRNA expression in single patched NG2-glia from PDGFRα-creERT; Rosa26-mGFP transgenic mouse hippocampus at postnatal 6-8 weeks in Figure 1d.

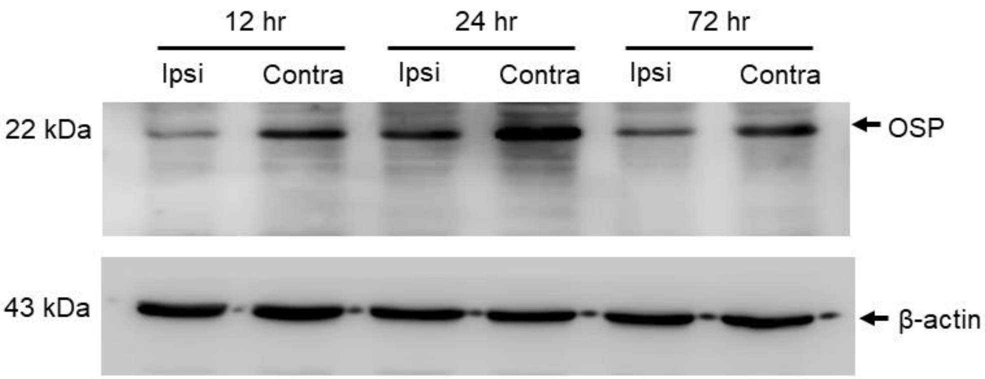

Supplementary Figure 9. Full-sized scan of Western blots in Figure 4c.

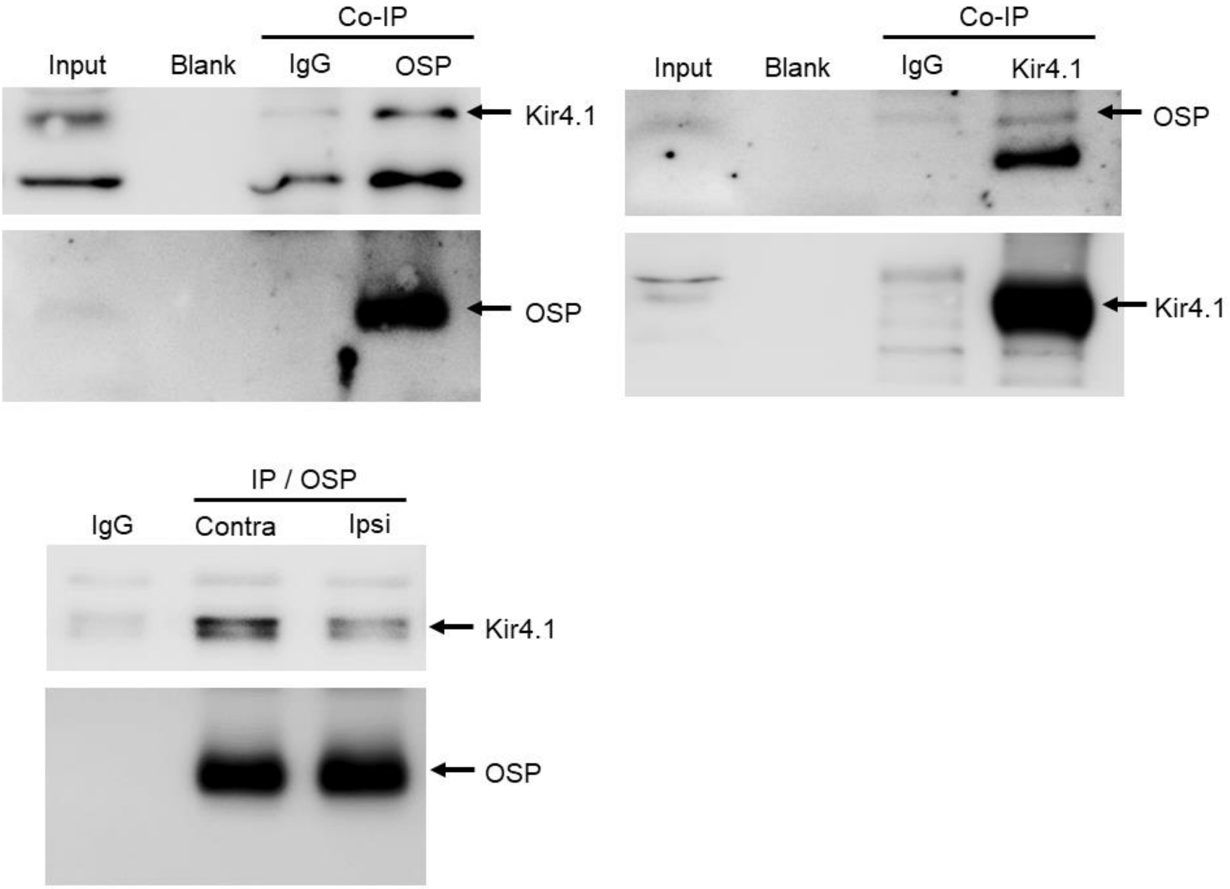

Supplementary Figure 10. Full-sized scan of Co-immunoprecipitation in Figure 4f.

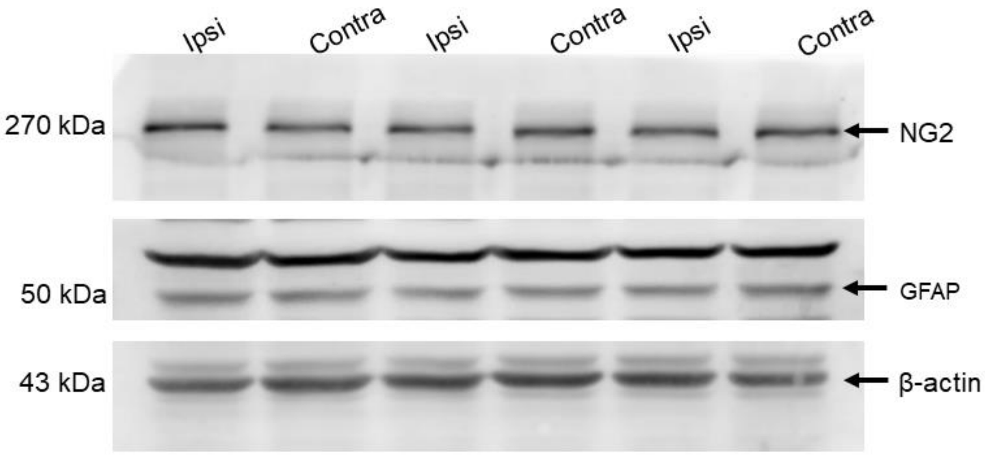

Supplementary Figure 11. Full-sized scan of Western blots for NG2 and GFAP expressions in both contralateral and ipsilateral hippocampus after 30 min tMCAO mice with 24 h reperfusion respectively at postnatal 8 weeks in Supplementary Figure 3a.

Supplementary Table 1. RNA-seq determination of inwardly rectifying K^+ channel genes in NG2-glia.

Gene name	Value [$\log_{10}$ (TPM + 1)]
Kcnj13	2.47
Kcnj10	1.80
Kcnj8	1.63
Kcnj3	1.46
Kcnj9	1.16
Kcnj16	1.06
Kcnj4	0.46
Kcnj12	0.43
Kcnj6	0.29
Kcnj11	0.18
Kcnj2	0.18
Kcnj15	0.01
Kcnj1	0
Kcnj14	0
Kcnj5	0
